# Unraveling the resistance mechanism for *Shigella* under stress of heavy metal Pb(II)

**DOI:** 10.1128/spectrum.01255-25

**Published:** 2025-12-02

**Authors:** Lihong Zhang, Baoyan Guo, Yingjie Li, Bingying Chen, Xueyong Zhou

**Affiliations:** 1 School of Life Science, Shanxi Engineering Research Center of Microbial Application Technologies, Shanxi Normal University 47842, Taiyuan, Shanxi, China; Università degli Studi di Napoli Federico II, Naples, Italy

**Keywords:** *Shigella*, Pb(II) resistance, XRD, FITR, biosorption

## Abstract

Lead is considered a main pollutant that is found in air, soil, and water, which is a dangerous waste and extremely toxic to any living organism. The use of micro-organisms is the most successful approach to remediate heavy metals from the environment due to their efficacy and financial viability. *Shigella* sp. D5 was isolated from a mining soil, which was found to be well capable of removing the heavy metal Pb(II). In the present research, biosorption of Pb(II) from aqueous solution via D5 living mass was described. Factors affecting biosorption efficiency, such as adsorbent dose, initial lead concentration, temperature, pH, and contact time, were investigated. The optimum levels of pH, initial Pb(II) concentration, temperature, and contact time were 7, 200 mg/L, 35°C, and 24 h, respectively. Under the optimized adsorption conditions, the removal rate of lead reached 82% when the initial concentration of lead was 50 mg/L. Both Langmuir and Freundlich isotherm models were fitted well with the equilibrium experimental data. The kinetic results showed that the sorption data closely followed a pseudo-second-order kinetic model. The micro-adsorption mechanism of Pb(II) was investigated by SEM, FTIR, XRD, and XPS techniques. It demonstrated that the anionic components like phosphate, oxhydryl, and chloride groups can remove Pb(II) through strong surface complexation and electrostatic attraction for D5. The strain D5 could transfer lead ions into highly insoluble compounds Pb_5_(PO_4_)_3_OH and Pb_5_(PO_4_)_3_Cl on the bacteria surfaces. All results indicated that it was feasible to effectively remove Pb(II) by using the *Shigella* sp. D5. This study elucidates the adsorption properties and resistance mechanism of *Shigella* for Pb(II).

**IMPORTANCE:**

Microbially mediated remediation is a promising approach for heavy metal pollution control due to its cost-effectiveness, environmental compatibility, and adaptability. Understanding the mechanisms of microbial adsorption and resistance to heavy metal is critical for guiding practical bioremediation efforts. In this study, we isolated a highly lead-resistant *Shigella* sp. D5 from coal mine-contaminated soil. Although *Shigella* are frequently detected in heavy metal-polluted environments, their adsorption capacities and the resistance mechanism to Pb(II) remain poorly characterized. Our findings advance the understanding of microbial strategy for heavy metal remediation and highlight their role in heavy metal mineralization processes.

## INTRODUCTION

Lead (Pb) is a heavy metal (HM) belonging, along with chromium (Cr) and cadmium (Cd), to the group of the first hazard class for the environment and humans (1, 2). Pb is carcinogenic and shows toxicity even at trace amounts, posing threats to environmental ecology and human health. Li et al. reported that Pb and Cd contributed to the greatest health risk, and leafy vegetables tended to be more contaminated than non-leafy vegetables from the crops/vegetables grown on smelting of nonferrous metals contaminated soil in Hunan Province, China (3). Xu et al. pointed out that relatively few investigations had explored the mechanisms of Pb(II) removal. Further, fewer papers studied the removal mechanisms of Pb(II) from Pb-containing minerals (4). Pb accumulation caused oxidative stress due to excessive reactive oxygen species (ROS) production in fish tissues. Oxidative stress by Pb exposure induced synaptic damage and neurotransmitter malfunction as neurotoxicity in fish (5). Lead contamination could negatively affect their liver and gut for birds, and heavy metal, especially Pb, may pose serious health risks to birds through the “gut–liver axis” (6). Pb caused neurocognitive impairment has been extensively studied (7).

*Shigella* spp. were reported in different heavy metal contamination environments in lots of literature (8–17). Some studies indicated the bioremediation potential of various *Shigella* isolates. Sultan et al. observed that *Shigella* sp. WG4 could resist mercury, silver, and arsenic sourced from Dal and Wular Lake of Kashmir valley in India (18). Noman et al. reported that the *Shigella flexneri* SNT22, the copper-resistant strain isolated from the wastewater-contaminated soil, could synthesize green copper nanoparticles (CuNPs), which could alleviate the Cd toxicity in plants (19). Lin et al. reported that the main Cr-bioreducing bacteria included *Trichococcus*, *Escherichia-Shigella*, and *Lactobacillus* (20). Elarabi et al. reported that the *S. flexneri* FACU was obtained with higher lead resistance (2500 ppm C_4_H_6_O_4_Pb), which was isolated from the industrial wastewater from different heavy metals contaminated locations in Egypt (21). Up to now, the mechanism of *Shigella* resistance to heavy metals is still unclear.

The objectives of this study were (i) to isolate Pb-resistant bacteria from peri-mining soils using culture-dependent methods, (ii) to determine the adsorption properties of Pb(II) by the bacteria, and (iii) to elucidate the mechanism of *Shigella* resistance to lead.

## MATERIALS AND METHODS

### Soil collection

The surface soils (0–20 cm) were obtained from agricultural and forested areas within 1 km of the Antaibao coal mine (112.43°E, 39.47°N; operational since 1985), located in Shuozhou City, Shanxi Province, China. The soil samples were immediately packed in sterile polyethylene bags, maintained on ice during transport, and stored at 4°C ± 1°C until analysis.

### Screening and isolation of Pb(II)-resistant bacteria

The lead nitrate (analytical grade) was dissolved in distilled water to prepare a 10 g·L⁻¹ Pb(II) stock solution, filtered through a 0.22 µm membrane, and stored at 4°C up to 7 days. The soil (10 g) and sterile water (90 mL) were mixed in a flask, shaken (170 rpm, 30°C, 2 h), settled for 30 min, then serially diluted and spread on nutrient broth (NB) solid medium (beef extract powder 3 g L^−1^, peptone 10 g L^−1^, NaCl 5 g L^−1^, and agar 15 g L^−1^, pH 6.5) with 100–1,000 mg/L Pb(II). After a 24-hhour incubation at 30°C, bacterial cells in NB medium were subjected to purification twice. The purified isolates were preserved on NB slants and in the refrigerator (−80°C), respectively.

### Morphological characterization and biochemical tests

The isolate was observed by the scanning electron microscopy (JSM-7600F, JEOL Ltd., Tokyo, Japan). Gram staining was carried out according to Harrigan et al. (22). Tests for indole production, gelatine liquefaction, starch hydrolysis, and hydrogen sulfide production were performed according to Smibert and Krieg (23). The ability to use different sugars was determined using phenol red broth media (cat. no. P0882, Amresco, USA) supplemented with 2% carbohydrate. Basal medium was used as the negative control. Three independent replicates were performed for each experiment.

### Identification of Pb(II)-resistant strain

The isolate was grown on NA plates (30°C, 10 h) and sent to Tsingke Biotech for DNA extraction (24). The protocol included liquid nitrogen-assisted cell lysis and Trelief kit-mediated DNA purification (TSP102-200). The 16S rDNA gene sequence of the isolate was amplified with the universal primer 27F (5′-AGAGTTTGATCCTGGCTCAG-3′) and 1492R: (5′-TACGGCTACCTTGTTACGACTT-3′). The amplification programs were used with the following parameters: 2 min at 98°C, 38 cycles × (10 s 98°C, 15 s 55°C, 15 s 72°C), 5 min at 72°C. The obtained sequence was compared with the higher similarity sequences from the NCBI database. The sequence alignments were generated in Clustal X 1.83 and MEGA 5.0 (25). A distance matrix method, including clustering by neighbor-joining (26) was used for phylogenetic analysis.

### Determination of the effect of lead on bacterial growth

Bacterial suspensions (1 mL) in mid-exponential growth phase were inoculated into 9 mL aliquots of nutrient broth (NB) liquid medium (beef extract powder 3 g L^−1^, peptone 10 g L^−1^, NaCl 5 g L^−1^ dissolved in 1 L ddH_2_O) supplemented with differential Pb(II) concentrations (0, 100, 200, 300, and 400 mg/L). The absorbance measurements at 600 nm wavelength were performed with a T-UV759 spectrometer (INESA, China) to track growth curves.

### Batch adsorption experiments

Batch removal studies were used to evaluate the Pb(II) removal of the isolate. The 10 g/L Pb(II) stock solution was prepared, and 2 g of biosorbent was supplemented. Initial Pb(II) concentrations were established from 50 to 250 mg/L. The pH was maintained at 5.5–7.5 using 0.1 M HCl/NaOH titration. Additionally, the effect of adsorbent dose (0.01–5.15 g/L) and contact time (0.5–48 h) was also estimated. After filtration with a 0.45-µm membrane, the metal concentration in the mixture was determined by the atomic absorption spectrometry (AAS).

### Heavy metal sorption by isolates

The biosorption capacity of the isolate for Pb(II) was quantitatively assessed using NB cultivation systems. Following autoclave sterilization (121°C for 25 min), 1 mL aliquots of log-phase bacterial suspension (OD600 = 0.5–0.8) were inoculated into 9 mL sterile medium. The Pb(II) solutions were filter-sterilized (0.22 µm) and dispensed into sterile liquid media. Cultures were incubated with shaking (170 rpm, 30°C), with samples periodically collected for analysis. All the experiments were performed in triplicate.

Culture broth was transferred into 50 mL conical tubes and centrifuged (6,000 rpm, 10 min). Supernatants were stored at 4°C for subsequent analysis of residual Pb²^+^ by AAS. The pelleted biomass was washed three times with ddH₂O, followed by oven-drying (80°C, 18 h) and then weighed.

The adsorbed heavy metal ions onto isolate biomass and removal percentage were calculated by equations 1 and 2, respectively.


(1) $$q_{e}=\frac{\left(C_{0}-C_{e}\right)V}{m}$$

where *q_e_* was the adsorption capacity of bacteria for heavy metal (mg g^−1^) at equilibrium, *C_0_* was the initial concentration of heavy metal (mg L^−1^), *C_e_* was the equilibrium concentration of heavy metal (mg L^−1^), *V* (L) was the volume of aqueous medium, and *m* was the dry weight (g) of isolate biomass (27). The suspensions were diluted to obtain a range of concentrations, i.e., 10 to 10,000 times. The concentration of heavy metals was quantified in the final dilution using atomic absorption spectrophotometry.


(2) $$Removal\;rate\;\left(\%\right)\;=\frac{C_{0}-C_{e}}{C_{0}}\times 100$$

where *C_0_* and *C_e_* were the initial and final concentrations of lead, respectively.

### Adsorption isotherms

Various models were adopted to understand the adsorption mechanism of metal ions present in aqueous solution (27). The experimental data of Pb(II) were subjected to linear regression analysis to identify suitable kinetic models. The coefficient of determination (*R^2^*) for each model was calculated and used for further evaluation. The following equations were adopted to generate the isotherm model for lead metal adsorption, the Langmuir and Freundlich models employed as empirical isotherm modes were expressed as equations 3 and 4, respectively.


(3) $$q_{e}=\frac{Q_{m}K_{L}C_{e}}{1+K_{L}C_{e}}$$

(4) $$q_{e}=K_{F}\;C_{e}^{\;1/n}$$

where *q_e_* and C_e_ were the same in equation 1, *Q*_*m*_ was the maximum adsorption capacity of isolation (mg/g), *K_L_* was the Langmuir constant related to the sorption strength (L/mg), *K_F_* was the Freundlich constant related to the adsorption capacity [(mg g^−1^) (L mg)^−1/*n*^], and *n* was the Freundlich indicating the adsorption intensity (dimensionless) (28, 29).

The pseudo-first-order and pseudo-second-order were employed to study the adsorption kinetics and expressed as equations 5 and 6, respectively.


(5) $$q_{t}=q_{e}\left(1-e^{-k_{1}t}\right)$$

(6) $$q_{t}=\frac{t}{\frac{1}{k_{2}q_{e}^{2}}+\frac{t}{q_{e}}}$$

where *q_e_* (mg/g) and *q*_*t*_ (mg/g) were the concentration of adsorbed heavy metal ion at equilibrium and at time *t*, respectively, *t* was the shaking time (h), and *k*_1_ and *k*_2_ were the adsorption rate of the first order and second order, respectively.

### Adsorption thermodynamics

The standard Gibbs energy (Δ*G°*), enthalpy (Δ*H*^0^), and entropy (Δ*S*^0^) changes give useful information regarding the disorder of the adsorption system, spontaneity, and feasibility of adsorption.


(7) $$\Delta G^{\circ }=-RT\ln K^{\circ }$$

(8) $$\Delta H^{0}=-\text{slope}\times R$$

(9) ΔS=intercept×R

where *K*^0^ is the standard equilibrium constant at adsorption equilibrium, dimensionless; Δ*G*° (kJ·mol^−1^) is the standard Gibbs free energy change, Δ*H*^0^ (kJ·mol^−1^) is the standard enthalpy change, Δ*S*^0^ (J·mol^−1^·K^−1^) is the entropy change, and *R* (J·mol^−1^·K^−1^) is the gas constant value 8.314.

### Mechanism of Pb(II) removal by the isolate

#### Scanning electron microscopy (SEM) observation

In order to decipher the changes in cell morphology due to Pb accumulation, SEM study was performed with the overnight grown bacterial cells in 50 mg/L, 150 mg/L compared with a control (devoid of Pb). The preparation of bacterial samples for SEM was done according to Bossù et al. (30). Cells were harvested by centrifugation at 10,000 rpm for 10 min after 24-h incubation at 30°C, then were washed thrice with PBS (pH = 7.2) and resuspended overnight in 2.5% glutaraldehyde (wt/vol in PBS, pH = 7.2) at 4°C. Cell pellets were washed again with PBS followed by dehydration with ethanol gradients. Three samples (control and two Pb-treated) were subsequently mounted in the gold grid and observed under SEM.

#### Fourier transform infrared spectroscopy (FTIR) analyses

FTIR studies were investigated using 1 mg samples in strained cells before and after Pb(II) adsorption. Surface functional groups were conducted by FTIR spectrometer (Nicolet 5700) in the range of 4,000–300 cm^−1^.

### X-ray diffraction (XRD) and XPS analyses

For both cases, bacterial cells were grown in 50 mg/L Pb(II) and 150 mg/L Pb(II) with NB liquid medium at 30°C with 170 rpm. The mixture of bacterial cells and culture medium was centrifuged and then washed with sterile water (pH = 7.0). Finally, the bacterial cells were lyophilized (vacuum freeze dryer FD-1A-50+, BIOCOOL, China). Bacterial cells untreated with Pb(II) were processed in the same way as the control group. All samples were further detected by XRD and XPS for the extracellular Pb. XRD patterns were recorded with an X-ray diffractometer using a Cu Kα spectral line at 40 kV and a 2θ range from 10° to 90° (Ultima IV, Rigaku Corporation, Japan). XPS spectra were obtained using an Al Kα spectral line by an X-ray photoelectron spectrometer (K-alpha+, ThermoFisher Scientific, American).

### Statistical analysis

All statistical analyses were performed using SPSS18. All the experiments were performed in triplicate. When the analysis of variance showed significant treatment effects, Tukey’s test (*P* < 0.05) was applied to make comparisons between treatments.

## RESULTS

### Growth and biological characteristics of Pb-resistant strain

**Fig 1:**
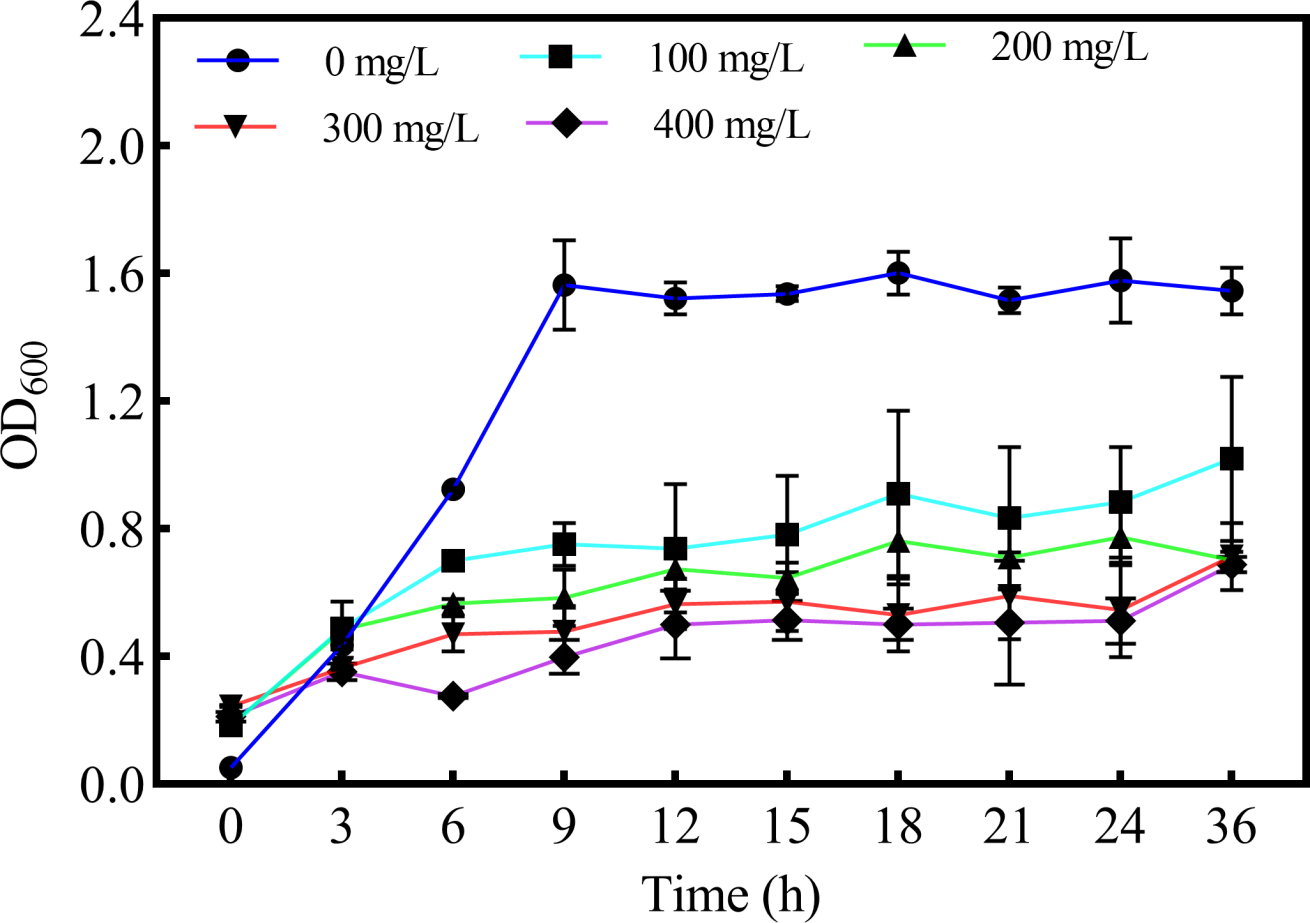
The growth curve of D5 in NB liquid medium with different Pb(II) concentrations.

To eliminate the influence of Pb(II) on the OD_600_ of bacteria, the bacteria were measured by washing with sterilized ddH_2_O. Fig. 1 showed the value of OD declined significantly under the Pb(II) stress compared with the control. Meanwhile, the cell concentrations were significantly reduced with the increase of Pb(II) concentration.

The cells were Gram-negative, rod-shaped, singular, 0.5–0.7 μm × 0.9–1.2 µm in size (Fig. 5A). The colonies were circular, wet, and smooth with regular edges, and the colonies were opaque and creamy on NB solid medium. The optimum temperature for growth was 35°C, and the pH range was between 5.5 and 7.5. Subcultivation for physiological and biochemical analyses was performed on NB medium at 35°C for 24 h (Table S1). Analysis of the carbon substrate utilization for growth of D5 demonstrated that the isolate D5 utilized the following substrates: maltose, glucose, sucrose, mannitol, fructose, tartrate, and starch. The isolate D5 did not show growth in inositol, rhamnose, and sodium pyruvate.

### 16S rRNA gene sequencing and phylogenetic analysis

**Fig 2:**
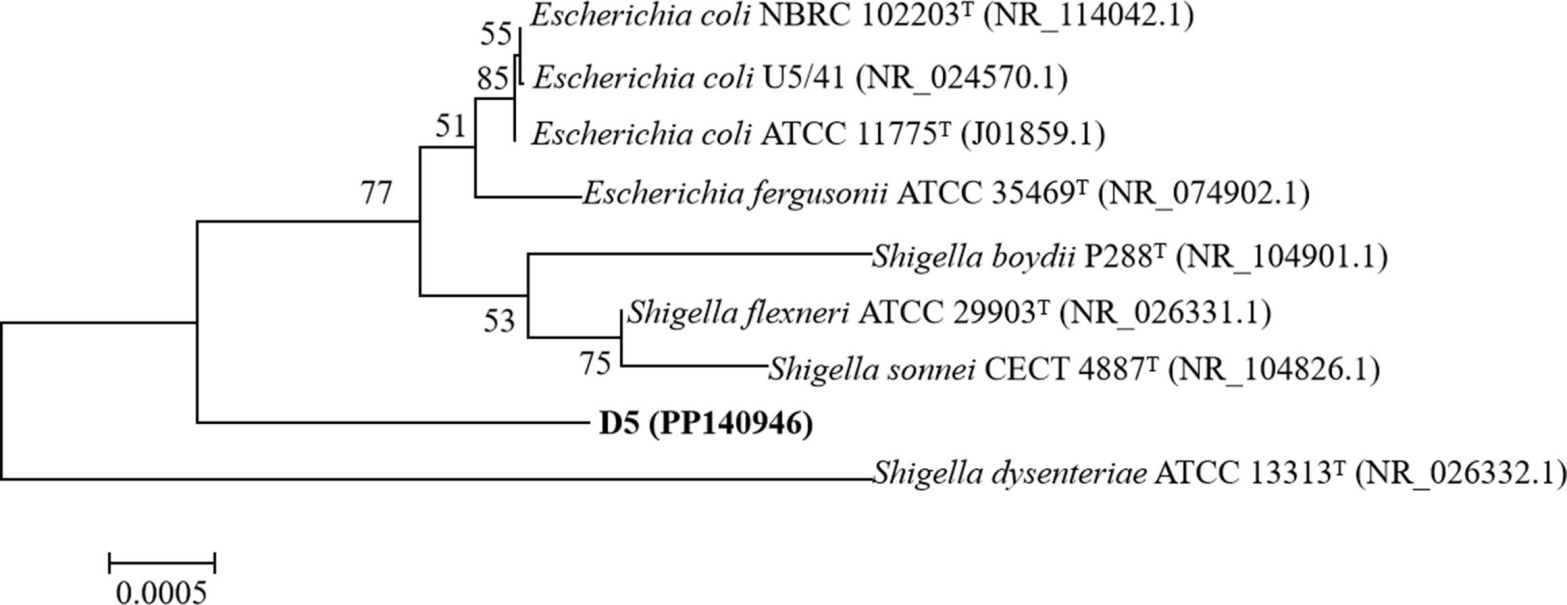
Phylogenetic tree constructed with 16S rRNA sequences available from the GenBank/EMBL/DDBJ database. The tree was constructed with MEGA 5.0 using the neighbor-joining with bootstrap values calculated from 1,000 trees based on 1,460 base pairs of the 16S rRNA sequences of the isolates D5 (GenBank accession number PP140946). The scale bar is shown in the figure.

Based on the 16S rRNA gene sequence phylogenetic analysis, the 16S rRNA sequences most closely grouped with *S. flexneri* (99.52% identity), followed closely by *Shigella sonnei* (99.38% identity); *Escherichia fergusonii* (99.38% identity); *Escherichia coli* (99.25% identity); *Shigella boydii* (99.11% identity); *Shigella dysenteriae* (99.11% identity). On the basis of the phylogenetic analysis, we concluded that the species was the *Shigella* sp. D5 (Fig. 2).

### Adsorption test

To investigate the Pb(II) absorption capacity of the isolate, different aqueous solutions with varying initial Pb(II) concentration, temperature, pH, inoculate dose, and contact time were tested.

**Fig 3:**
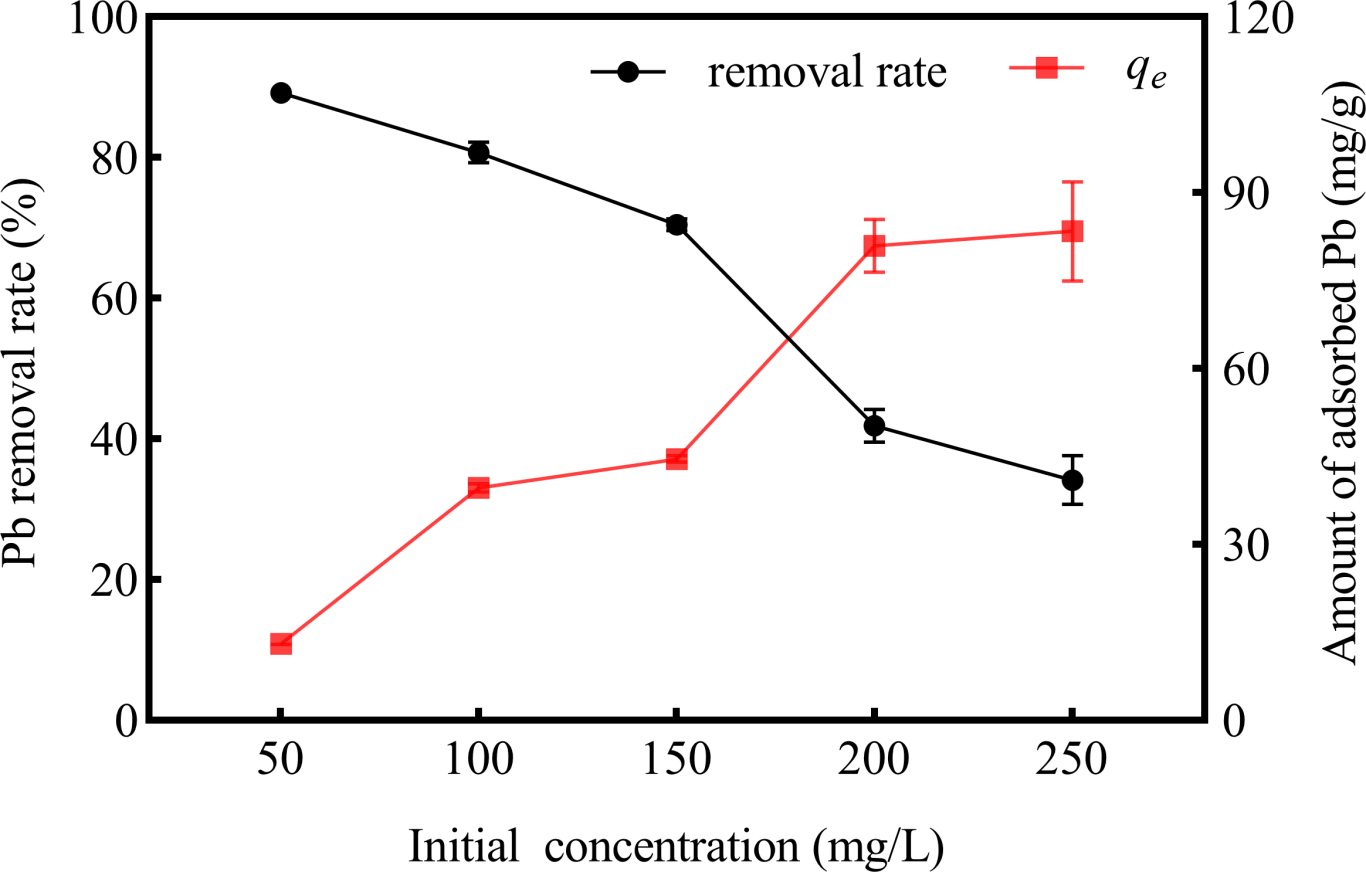
Effects of initial concentration of Pb(II) onto the strain D5 adsorption capacity (contact time of 24 h, 35°C, pH at 7).

The initial concentrations of Pb(II) had an obvious influence on the corresponding adsorptions (Fig. 3). With the concentration of Pb(II) ranging from 50 to 250 mg/L, the removal rate decreased gradually from 89% to 34%. However, the amount of adsorbed Pb(II) (*q*_e_) increased gradually from 13.01 to 83.41 mg/g.

The effect of adsorbent dose with a contact time of 24 h was shown. As the adsorbent dose was from 0.09 to 2.27 g/L, the removal rate of the Pb(II) showed almost linear growth. The maximum adsorption dose was 2.27 g/L (removal percentage 73%).

**Fig 4:**
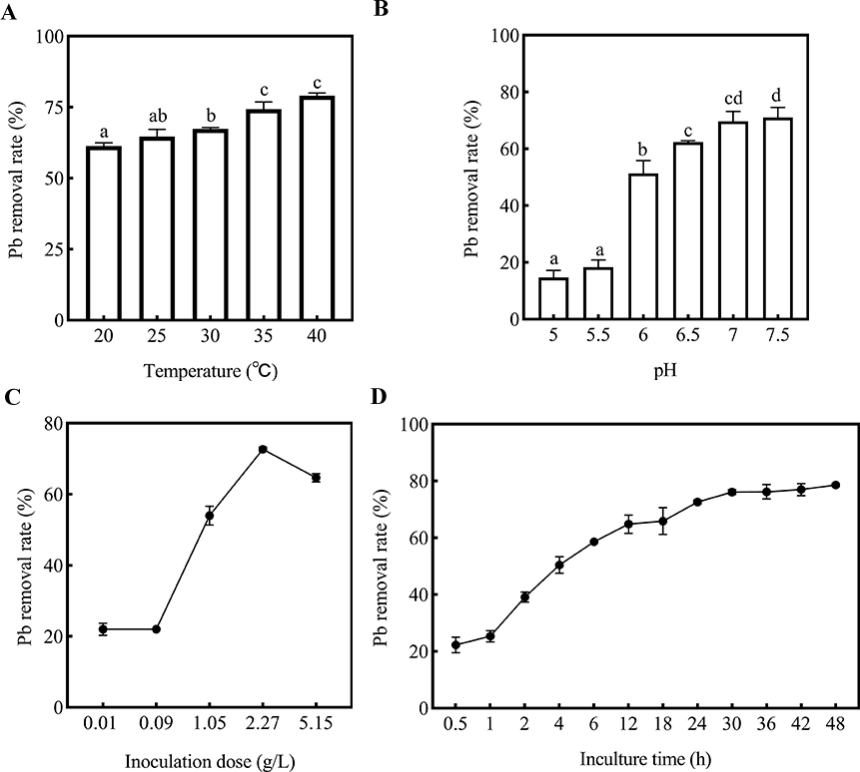
Influence of different conditions on the removal efficiency of D5. (**A**) Influence of temperature on the removal efficiency of D5. (**B**) Influence of pH on the removal efficiency of D5. (**C**) Influence of inoculate dose on the removal efficiency of D5. (**D**) Influence of contact time on the removal efficiency of D5 (200 mg/L pb(II), 30°C, pH = 7).

The effect of temperature of Pb(II) on the efficiency of adsorption was shown in Fig. 4A. The temperature had a certain impact on adsorption. As the temperature increased from 20°C to 40°C, the Pb removal rate increased from 61% to 79%. At 35°C, the strain D5 played better adsorption performance on Pb(II).

In a comprehensive evaluation of the effect of pH on the growth range of bacteria and lead precipitation, we set the pH ranging from 5.0 to 7.5. As presented in Fig. 4B, with the increase in pH value, the adsorption capacity gradually increased from 17% to 68%. At pH 7.0, the strain D5 played better adsorption performance on Pb(II).

The effect of contact time of Pb(II) on the efficiency of adsorption was shown in Fig. 4D. The removal rates gradually increased from 22% to 79%. When the contact time was extended to 24 h, 73% was achieved, and then the adsorption capacity slowed down.

### Adsorption behaviors

#### Adsorption isotherm

The equilibrium isotherm is crucial to understand the interaction between D5 and Pb(II).

The Langmuir model, based on monolayer coverage (31), was fitted well to experimental data (*R*^2^ = 0.9769; Fig. S1). The maximum amount of ions sorbed by D5 was 520.09 mg g^−1^ (Table 1). The Freundlich model was fitted well to experimental data with a high correlation coefficient (*R*^2^ = 0.9738; Fig. S1). The Freundlich parameter of 1/*n* denotes the adsorption driving force, and it is generally considered that the value of n greater than one indicates good adsorption (32). Since the value of *n* for Pb(II) adsorption by D5 was 1.79 (Table 1), the above adsorption was favorable.

**TABLE 1:** Isothermal study for adsorption of Pb(II) by *Shigella* sp. D5

Langmuir isotherm model			Freundlich isotherm model		
*q*_*m*_ (mg/g)	*K*_*L*_ (L/mg)	*R* ^2^	*K*_*F*_ (mg g^−1^)(L mg^−1/*n*^)	1/*n*	*R* ^2^
520.09	0.0022	0.9769	1.712	0.8636	0.9738

The value of *n* for Pb(II) adsorption by D5 was 1.79. It is generally considered that values of *n* greater than one indicate good adsorption occurs naturally (32). The Freundlich model was also suitable for explaining the lead biosorption processing of the strain D5 (Fig.S1; Table 1). It meant the uptake mechanism of Pb(II) was based on a chemical reaction and a physical adsorption process for D5.

#### Adsorption kinetic

Kinetic models were used to describe the mechanism of the biosorption process. The pseudo-first-order and pseudo-second-order models were used in this work (31). The equations presented in Fig. S2 and Table 2 were used for explaining the experimental data. According to the correlation coefficients, the kinetic model revealed that the pseudo-second-order with its high correlation coefficients (*R*^2^ = 0.9900) demonstrated the validity and superiority of the second-order model than the pseudo-first-order model. Based on the results, it can be concluded that the pseudo-second-order model is better to describe Pb(II) removal by D5 adsorption.

**TABLE 2:** Kinetic parameters for the biosorption of Pb(II) onto D5

Pseudo-first-order kinetics			Pseudo-second-order kinetics		
*k*_1_ (h^−1^)	*q*_*e*1_ (mg g^−1^)	*R* ^2^	*k*_2_ (g mg^−1^·min^−1^)	*q*_*e*2_ (mg g^−1^)	*R* ^2^
7.67 × 10^−2^	30.9187	0.7932	3.988 × 10^−3^	81.4995	0.9900

The experimental data of the adsorption process were well fitted with the pseudo-2nd order kinetics, which indicated that Pb(II) adsorption was a chemisorption process on heterogeneous surfaces of strain D5.

#### Adsorption thermodynamic

The standard enthalpy change (Δ*H*⁰) for the adsorption of lead ions by D5 cells was calculated as 3.714 kJ/mol. The positive Δ*H*⁰ value indicates an endothermic process. The entropy change (Δ*S*^0^) was 119.607 J/mol. The positive Δ*S*^0^ value signifies an increase in system disorder after adsorption equilibrium, confirming that adsorption is an entropy-increasing process. The calculated Gibbs free energy change (Δ*G*^0^) values were consistently negative, demonstrating that the reaction is spontaneous under the experimental conditions (Table 3; Fig. S3).

**TABLE 3:** Thermodynamic parameters of Pb^2+^ adsorption by D5

Initial Pb^2+^ concentration (mg/L)	Δ*H*^0^ (kJ/mol）	Δ*S*^0^ (J/[mol·K])	Δ*G*^0^ (kJ/mol)				
			293	298	303	308	313
100	3.714	119.607	−0.796	−0.841	−1.394	−2.347	−3.062

### Removal mechanisms of Pb(II) for *Shigella* sp. D5

#### Surface morphology observation of D5

**Fig 5:**
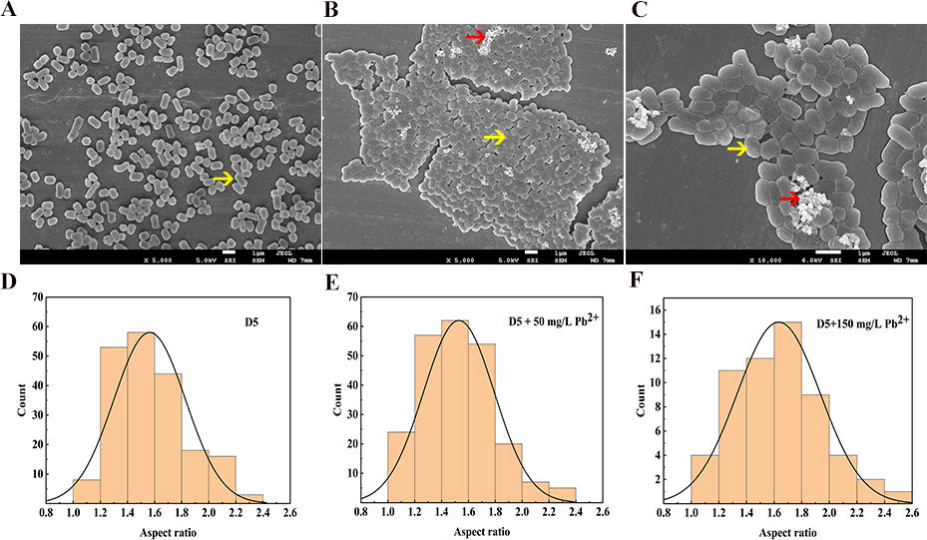
Scanning electron microscopy (SEM) images of the D5 in the absence and presence of Pb(II) (50, 150 mg/L) revealed changes in cell surface morphology. (**A**) 0 mg/L Pb(II); (**B**) 50 mg/L Pb(II); (**C**) 150 mg/L Pb(II); (**D–F**) a histogram showing the aspect ratio distribution of D5. The bacterium was incubated for 24 h under 30°C and 170 rpm. The samples for SEM analysis were collected at 24 h, and the red arrow: bright spots; the yellow arrow: the strain D5.

The morphology of extracellular Pb-containing minerals formed by D5 was observed under SEM (Fig. 5).

When the strain was cultured without Pb in the environment, the D5 showed a good dispersion, and no adhesion was observed between the cells (Fig. 5A). When it was exposed to the 50 and 150 mg/L Pb(II), the bacterium gathered together, and the edge of cells was not clear. Meanwhile, the insoluble precipitates aggregated to form a white floc (Fig. 5B and C), which was the Pb(II)-induced response for D5. We inferred the strain D5 gathered together to form biofilms under the lead stress, which may accentuate their remediation efficacy. Amrita and Rina pointed out that bacterial cells generally respond to metal stress by biofilm formation and maintenance (33). Rod-shaped bacterial cells can readily adapt their lengths and widths in response to environmental changes (34). The results showed that as the lead concentration increased in the environment, the proportion of cells with the aspect ratio η (length/width) of 1.6–1.8 increased (Fig. 5D through F). In addition, at initial lead concentrations of 0, 50, and 150 mg/L, the bacterial coverage rates were 26.04%, 64.87%, and 48.56%, respectively.

#### Analysis of a Fourier transform infrared spectroscopy (FTIR) study

**Fig 6:**
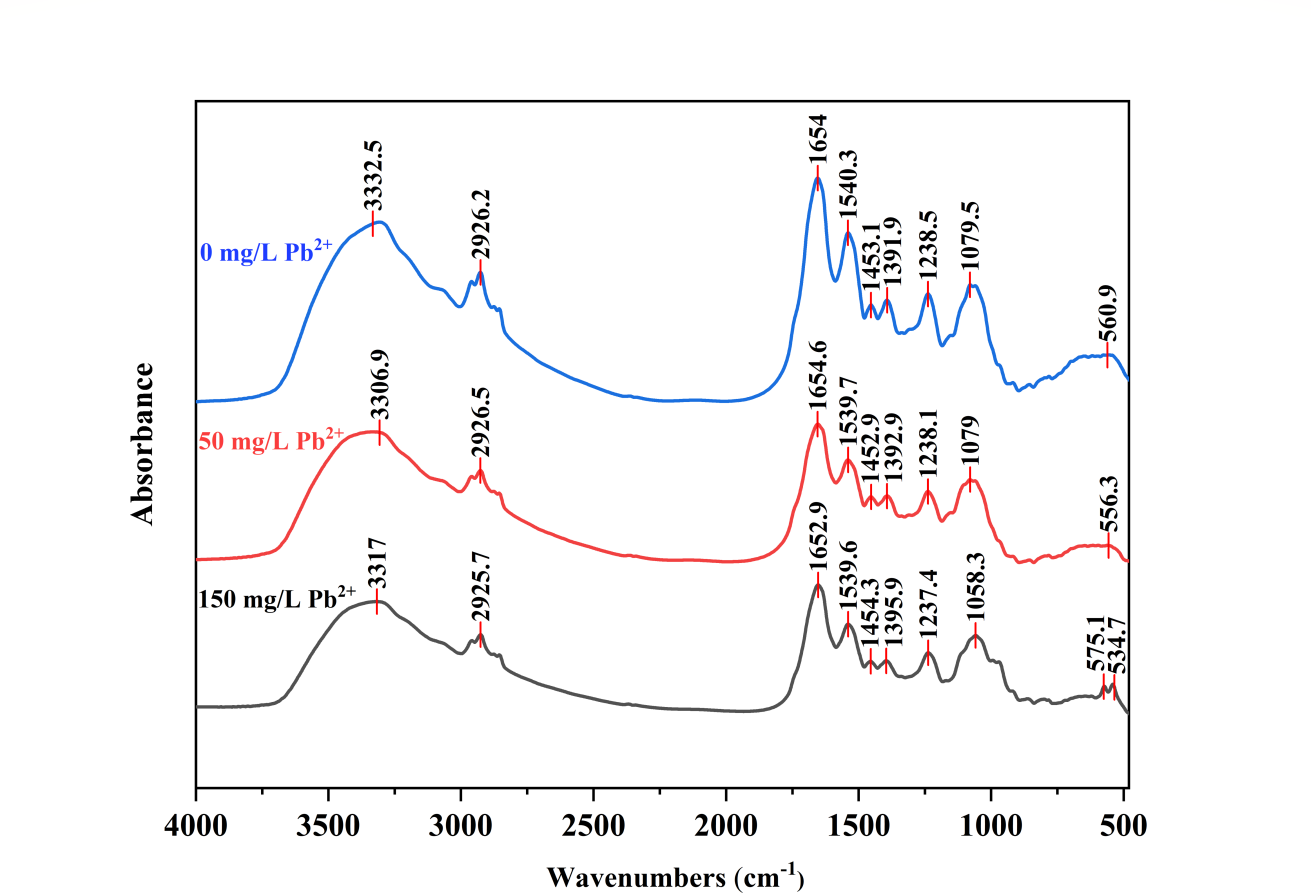
FTIR spectrum of the strain D5 before biosorption and after Pb(II) biosorption. All samples were incubated under 30°C, 170 rpm, 24 h.

The functional groups associated with Pb(II) biosorption were characterized using FTIR spectroscopy under varying Pb(II) concentration conditions (0, 50, and 150 mg/L) (Fig. 6). Significant changes in band intensities were observed in the FTIR spectra of D5 following interaction with Pb(II). Fig. 6 shows the presence of broad and strong bands at 3,000–3,600 cm^−1^ corresponding to hydroxyl groups (-OH). The peak at 850–1,500 cm^−1^ corresponding to Phosphorus-oxy compounds (*P* = O, P-OH, and PO_4_^3-^). The band shift from 560.99 to 556.30 (50 mg/L) or to 573 and 541.7 cm^−1^ (150 mg/L) was assigned to C-Cl group involvement (35).

The FTIR results confirmed the involvement of phosphate, chloride, and hydroxyl groups present in the bacterial cell wall for Pb binding.

#### X-ray diffraction (XRD)

**Fig 7:**
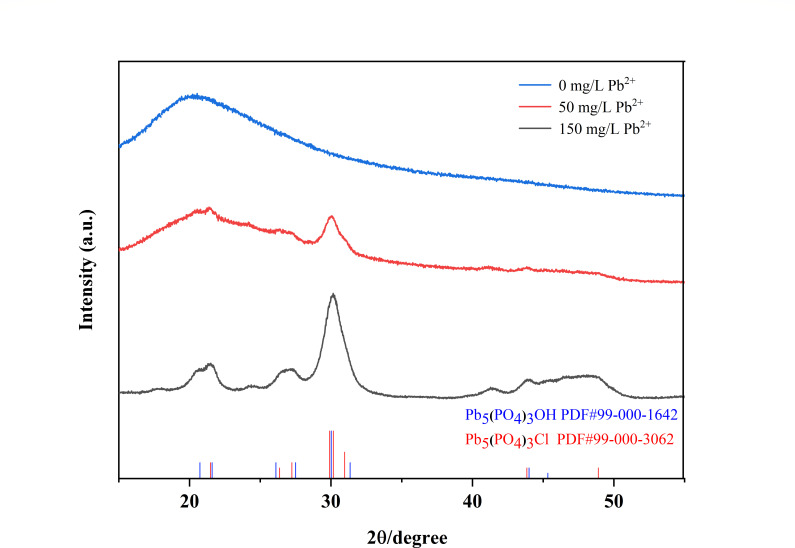
XRD pattern of extracellular Pb-containing products. All samples were incubated under 30°C and 170 rpm at 24 h for XRD analysis. Notes: 0 mg/L represented D5 without Pb(II); 50 mg/L represented D5 with 50 mg/L Pb(II); 150 mg/L represented D5 with 150 mg/L Pb(II); blue arrow: Pb_5_(PO_4_)_3_Cl; black arrow: Pb_5_(PO_4_)_3_OH.

The crystal structure and the composition of extracellular Pb-containing products by D5 strain under the lead press were detected by XRD technique. The XRD spectra have Pb_5_(PO_4_)_3_OH peaks at 2θ = 20.737°, 21.604°, 26.111°, 27.507°, 30.023°, and 31.362°, respectively, which is in agreement with the (99-000-1642) standard card from Jade 9. In addition, the XRD spectra have Pb_5_(PO_4_)_3_Cl peaks at 2θ = 21.488°, 26.371°, 29.909°, 30.178°, 30.972°, and 27.241°, respectively, which is in agreement with the (99-000-3062) standard card from Jade 9. As shown in Fig. 7, a broad peak centered around 2θ = 30° was observed in the samples with lead pressure. The peak arose from overlapping contributions, including a component at 30.023° and 31.362° attributed to Pb_5_(PO_4_)_3_OH and components at 29.909°, 30.178°, and 30.972° attributed to Pb_5_(PO_4_)_3_Cl. The XRD results revealed that the newly formed insoluble substance was Pb_5_(PO_4_)_3_Cl and Pb_5_(PO_4_)_3_OH on the cell wall. The XRD results were consistent with the FTIR spectra results. According to the FTIR pattern, phosphate, hydroxyl, and Cl groups were involved in Pb(II) binding. As the concentration of lead ions in the environment increased, the types of Pb-containing substances remained unchanged, while the content increased (Fig. 7). The finding was consistent with the results from Xu et al. (4) that Pb_5_(PO_4_)_3_Cl was reported on the cell wall for *Penicillium polonicum* to reduce to Pb inside the isolate cell (4).

#### XPS results

**Fig 8:**
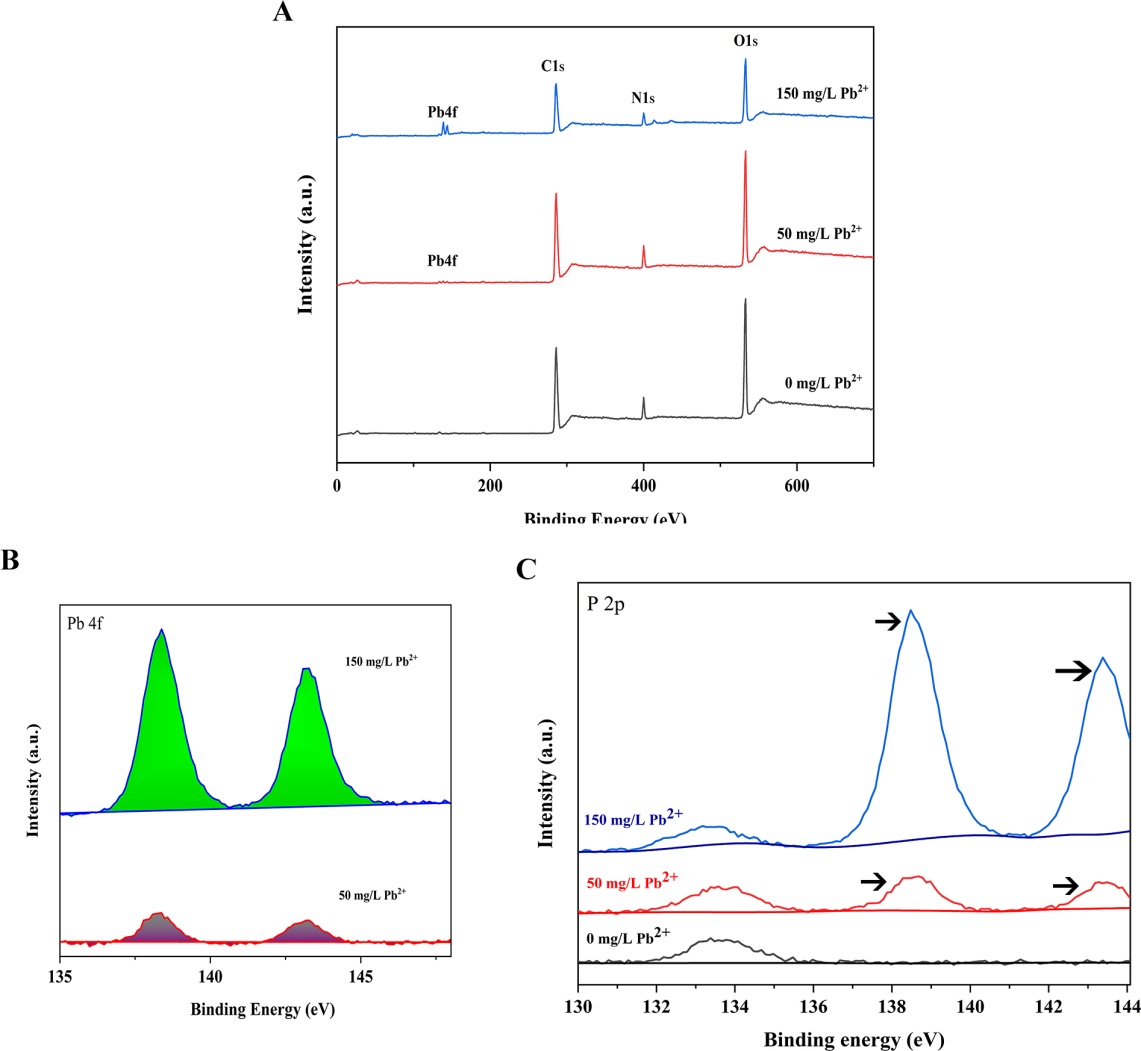
Survey XPS spectra of the D5 Pb-precipitant. (**A**) Survey XPS spectra of the D5 Pb-precipitant. (**B**) The survey Pb-XPS spectra of the D5 Pb-precipitant. (**C**) The survey P-XPS spectra of the D5 Pb-precipitant. The black arrows: the additional peaks of P2p. All peaks were referenced to the adventitious C1s peak at 284.8 eV.

In the XPS analysis, the samples with strain D5 and Pb(II) ion culture showed Pb 4f peaks at 138.38 and 143.28 eV (Fig. 8B), and the additional peaks of P2p appeared at 138.48 and 143.38 eV (Fig. 8C). Notably, the binding energies of the lead and phosphorus peaks were nearly identical, and their peak shape and peak ratio were similar. Moreover, the lead insoluble substances were significantly improved with the increase of Pb concentration in the environment (Fig. 8).

The elemental composition is as follows: C1s 66.28%, N1s 6.59%, O1s 26.8%, and P2p 0.32% in the control group; C1s 66.76%, N1s 6.18%, O1s 24.85%, and P2p 0.42% in 50 mg/L Pb(II) stress; C1s 65.55%, N1s 6.96%, O1s 24.59%, P2p 1.6%, and Pb4f 0.41% in 150 mg/L Pb(II) stress. The results revealed that under the Pb^2+^ stress, the concentrations of N and P elements increased significantly, while the concentrations of O significantly decreased compared with the control. We noticed that the percentage of Pb4f can be accurately quantified when the concentration was 150 mg/L Pb. All the results indicated that Pb(II) combined phosphorus and oxygen to form the new compound.

## DISCUSSION

Nature itself can mitigate the heavy metal pollution by employing the microorganisms, which can develop certain adaptive mechanisms for survival in heavy metal-infested sites (33). The Pb resistance mechanism from microbial impact including efflux and immobilization, siderophores, intracellular binding by metallothioneins, extracellular polymeric substances, and so on (1). However, the difference in adsorption behaviors and mechanisms of different microorganisms remains uncertain. Microbially induced phosphate precipitation (MIPP) was an effective and eco-friendly method for Pb(II) stabilization. The commercial *Bacillus subtilis* (BS), (CCTCC AB 98002) was a superior alternative for MIPP on Pb(II) stabilization, which could transfer the mobile Pb(II) into intra-, supra-, and extra-cellular Pb_5_(PO_4_)_3_Cl (36). Amer and Kim reported that *Delftia acidovorans* Pb11 and *Azonexus caeni* Pb2 could successfully utilize Pb(II) (37). The SEM-EDS and XPS analyses showed the isolates could form the Pb_3_(PO_4_)_2_, which was highly insoluble in water with very low solubility products. Lately, it had been widely reported that fungi could secrete oxalic acid and citric acid to precipitate Pb(II) ions and reduce Pb(II) toxicity. Xu et al. reported that Pb(II) was immobilized as lead oxalate outside the isolate cell, bound with phosphate, nitro, halide, hydroxyl, amino, and carboxyl groups on the cell wall, precipitated as pyromorphite Pb_5_(PO_4_)_3_Cl on the cell wall, and reduced to Pb(0) inside the cell for *Penicillium polonicum* (4). Montiel-Rozas et al. reported Pb was found on the cell surface in the form of PbS and PbCO_3_ through X-ray diffraction (XRD) for *Pleurotus ostreatus* ISS-1 (38). Qin et al. reported two phosphate-solubilizing bacteria with high Pb removal rates, *Klebsiella* sp. M2 and *Kluyvera* sp. M8 could induce the formation of Pb_2_(PO_4_)_4_, Fe_2_Pb_3_(PO_4_),_2_ and PbS precipitated that immobilized Pb in the solution (39). Yang et al. showed the iron-oxidizing strain *Zoogloea* sp. FY6 could simultaneously remove nitrate, tetracycline, and Pb(II). Meanwhile, the XRD result showed the PbO and PbO_2_ produced during co-precipitation (40). Chen et al. reported that combined forms of Pb mainly accounted for Pb-polysaccharides (Pb-OH of carbohydrates) in the cell wall for *Cladophora rupestris* (41). In our research, all results indicated that it was feasible to effectively remove Pb and P for *Shigella* sp. D5. The ability of D5 to reduce the bioavailability of environmental Pb made it potentially useful for bacteria-assisted phytostabilization of Pb-heavy-metal-contaminated environment. Although we have elucidated the lead resistance mechanisms from genus *Shigella*, expanding understanding of the resistant lead capabilities, the *Shigella* group remains an opportunistic pathogen posing health risks. Therefore, the application research in environmental remediation must address many aspects such as co-occurrence of resistance/virulence traits, ecological risks, and ethical issues.

### Conclusions

The genus *Shigella* was reported in different heavy metal pollution environments. However, to the best of our knowledge, few studies were available in the literature about heavy metal resistant characteristics and relevant resistant mechanisms. In our work, the analysis of Pb(II) adsorption capacity and adsorption kinetics for *Shigella* sp. D5 showed that the bacterium had a good Pb(II) removal ability. The equilibrium experimental data exhibited good fits to both Langmuir and Freundlich isotherm models. The sorption kinetics demonstrated close adherence to a pseudo-second-order model. The thermodynamic studies showed that the biosorption process is endothermic. The analysis of cell morphology changes and the types of adsorption products under the Pb stress indicated that the bacterium tended to gather together and possessed a relatively stable resistance ability by forming insoluble precipitates pyromorphite Pb_5_(PO_4_)_3_Cl and Pb_5_(PO_4_)_3_OH particles on the cell wall surface, which lowered the bioavailability and the toxicity of Pb(II) to the bacterium. The current research results indicated that this bacterium could better exert its ability to remediate pollution in environments rich in lead.

The present study provided valuable results regarding the use of *Shigella* strains for the efficient removal of heavy metals. Moreover, this study also provided a foundation for further understanding the mechanism of Pb(II) removal by bacteria and the biogeochemical cycle of Pb(II) on a microscopic scale.

## Data Availability

Some data used in the study were obtained from publicly accessible websites (ncbi.nlm.nih.gov).

## SUPPLEMENTAL MATERIAL

Supplemental material spectrum.01255-25-s0001.docx Table S1; Fig. S1 to Fig. S3.

ASM does not own the copyrights to Supplemental Material that may be linked to, or accessed through, an article. The authors have granted ASM a non-exclusive, world-wide license to publish the Supplemental Material files. Please contact the corresponding author directly for reuse.
