# Supplementary material for: Unraveling the resistance mechanism for Shigella under stress of heavy metal Pb(II)
Source: Microbiol Spectr. 2025 Dec 2;14(1):e01255-25. doi: 10.1128/spectrum.01255-25 (PMC12772253; doi:10.1128/spectrum.01255-25)
Supplement: Supplemental material — Table S1; Fig. S1 to Fig. S3. [file spectrum.01255-25-s0001.docx]

**Supplement material**

**Table S1** Morphological and biochemical characteristics of the isolate D5

| Characteristic | Isolate D5 |
| --- | --- |
| Shape^a^ | Rod |
| Size (μm) ^a^ | 0.5-0.7 × 0.9-1.2 |
| Flagella^a^ | - |
| Gram strain | Negative |
| Starch hydrolysis | + |
| Gelatine hydrolysis | - |
| Voges-Proskauer | - |
| H_2_S production | - |
| Methyl red test | + |
| Indole production | - |
| Use of carbon source |  |
| Inositol | - |
| Maltose | + |
| Glucose | + |
| Sucrose | + |
| Rhamnose | - |
| Mannitol | + |
| Fructose | + |
| Tartrate | + |
| Sodium pyruvate | - |
| Xylose | + |

^a^ Incubation in NB solid medium


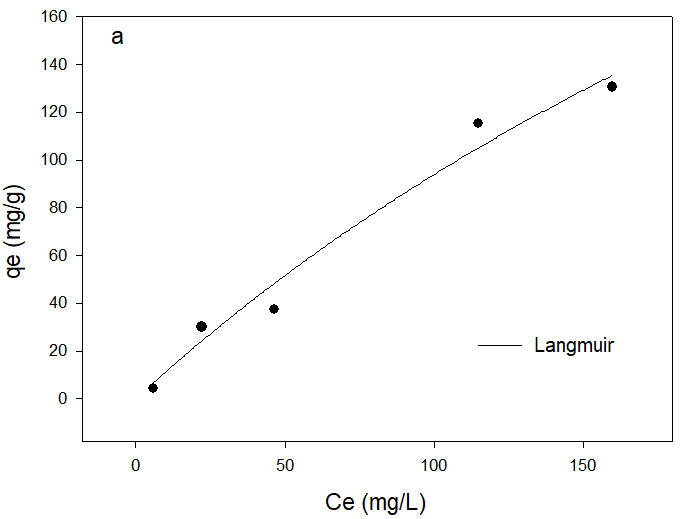

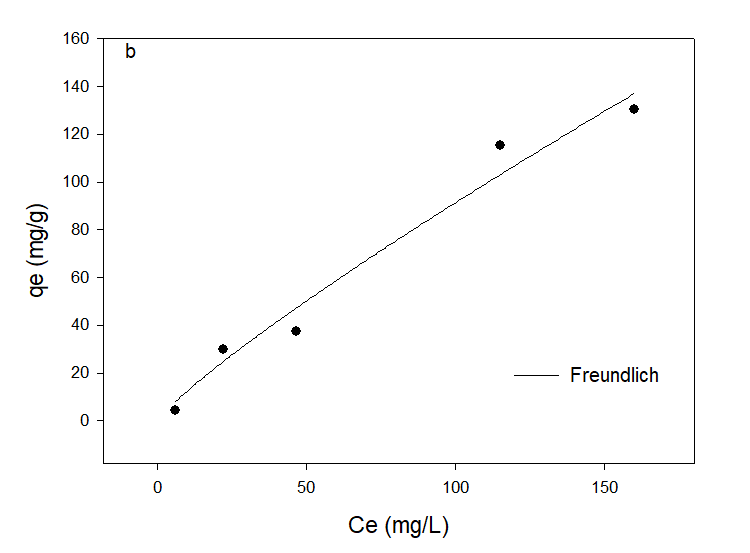


**Fig.S1.** Fitting parameters of Pb(II) adsorption curve (a) Langmuir model, (b)Freundlich model.


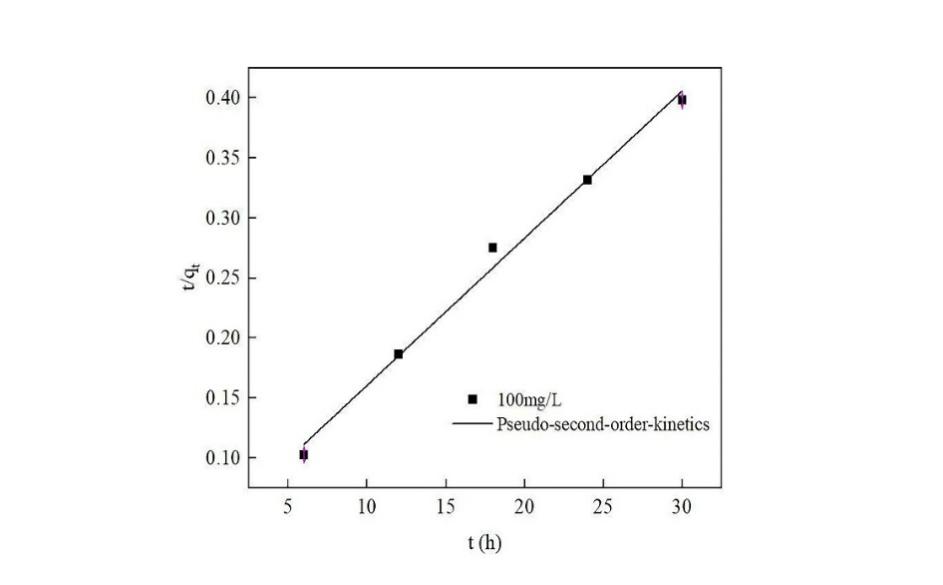

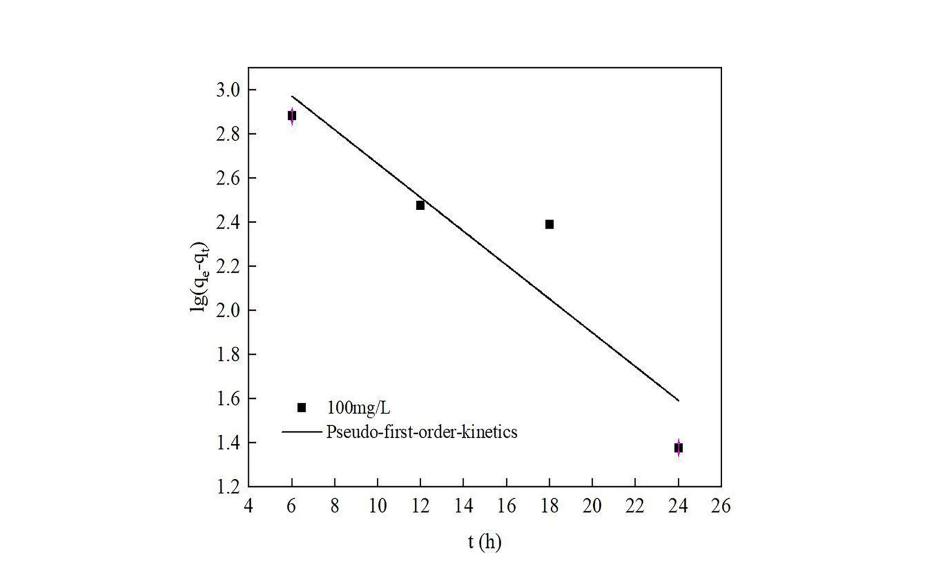


**Fig.S2.** Biosorption kinetics of Pb(II) from D5.


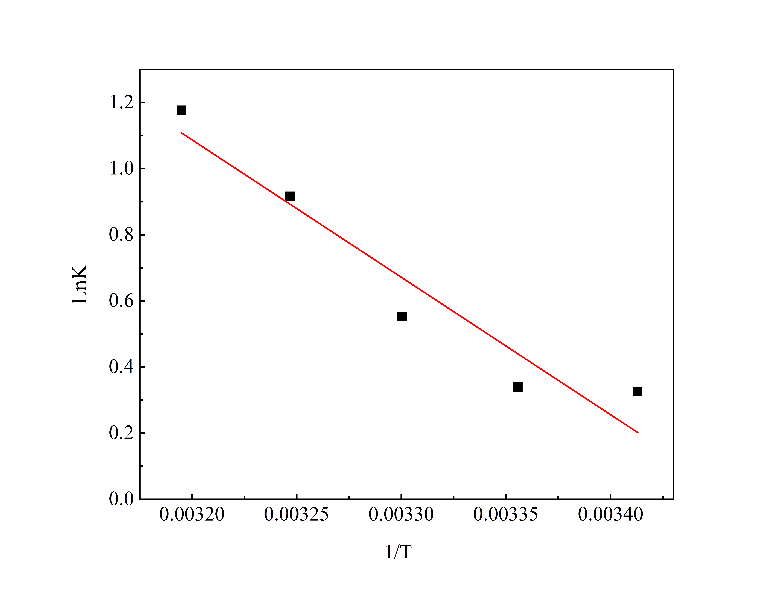


**Fig. S3.** The thermodynamic model of Pb(II) adsorption of D5
